# Factors influencing oviposition behaviour of the invasive pest, *Drosophila suzukii*, derived from interactions with other *Drosophila* species: potential applications for control

**DOI:** 10.1002/ps.7693

**Published:** 2023-08-08

**Authors:** Trisna Dewi Tungadi, Glen Powell, Bethan Shaw, Michelle T Fountain

**Affiliations:** ^1^ NIAB Cambridge UK; ^2^ School of Life Sciences Keele University Keele UK; ^3^ New Zealand Institute for Plant and Food Research Ltd Auckland New Zealand

**Keywords:** attraction, competition, Drosophilae, *melanogaster*, pheromone, repellence

## Abstract

*Drosophila suzukii* (Matsumura) or spotted wing *Drosophila* is a worldwide invasive pest of soft‐ and stone‐fruit production. Female *D. suzukii* lay their eggs in ripening fruit and the hatched larvae damage fruit from the inside, rendering it unmarketable and causing significant economic loss. Current methods to reduce *D. suzukii* population in the field primarily rely on chemical insecticides which are not a sustainable long‐term solution and increase the risk of resistance developing. Several studies demonstrate that when *D. suzukii* encounter or coexist with other *Drosophila* on a food source, this is usually a disadvantage to *D. suzukii*, leading to reduced oviposition and increased larval mortality. These effects have potential to be exploited from a pest management perspective. In this review we summarise recent research articles focusing on the interspecific interactions between *D. suzukii* and other *Drosophila* species aimed at understanding how this drives *D. suzukii* behaviour. Potential semiochemical and microbiome impacts are postulated as determinants of *D. suzukii* behaviour. Development of control practices focusing on reducing *D. suzukii* populations and deterring them from laying eggs by utilising factors that drive their behaviour are discussed. © 2023 The Authors. *Pest Management Science* published by John Wiley & Sons Ltd on behalf of Society of Chemical Industry.

## Graphical abstract

*Drosophila suzukii* or spotted wing Drosophila is a worldwide invasive pest of soft‐ and stone‐fruit production. Management primarily relies on chemical insecticides and sanitary practices. We review works on factors influencing interactions between *D. suzukii* and other Drosophila species, its effects on *D. suzukii* oviposition, and potential applications for control.

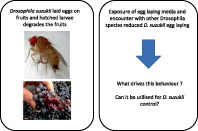

## 1 INTRODUCTION

*Drosophila suzukii* (Matsumura) (Diptera: Drosophilidae), also known as spotted wing *Drosophila*, is an invasive pest of soft‐ and stone‐fruit which has spread to most continents of the world.^1^, ^2^, ^3^, ^4^, ^5^, ^6^, ^7^ *Drosophila suzukii* is highly fecund with multiple generations per year,^8^, ^9^ has a wide host range, and can adapt to a range of climatic conditions which have contributed to its global spread and solidified its status as an invasive pest.^10^, ^11^, ^12^, ^13^

Female *D. suzukii*, unlike other *Drosophila* ^14^ have an enlarged and serrated ovipositor which enables them to pierce the skin of intact, ripening fruits to lay their eggs in (oviposit).^15^ Larvae feed on the fruit pulp, leading to fruit collapse and economic losses for growers.^16^ Oviposition wounds become entry points for secondary infection by fungal and bacterial pathogens and egg laying sites by other fruit flies.^3^, ^15^, ^17^, ^18^ Cultivar, fruit traits, and fruit ripening stage also influences fruit susceptibility to *D. suzukii* oviposition.^19^ Current methods to reduce *D. suzukii* populations rely primarily on field‐based and cultural approaches which require high labour input and are costly to growers.^20^, ^21^, ^22^ The repeated use of a limited selection of chemical insecticides increases the risk of insecticide resistance developing^23^, ^24^ and, as such, resistance to spinosad has been found in strawberry plantations in California^25^ and cherry orchards in Italy.^26^ The use of chemicals to suppress *D. suzukii* populations has negative consequences for the control of other key pests, such as western flower thrips, *Frankliniella occidentalis* (Pergande), which were successfully managed with augmented releases of predators prior to the arrival of *D. suzukii*.^27^ Biological control measures to manage *D. suzukii* populations have been evaluated with varying reports of efficacy.^28^ Often, the costs associated with the volume of predators, parasitoids, and biological control agents required to prevent yield loss exceeds the value of economic losses in the absence of these methods.^28^, ^29^ Development of push–pull systems utilising semiochemical attractants and repellents to drive *D. suzukii* away from the crop has been explored.^30^, ^31^, ^32^ Chemical repellents to reduce *D. suzukii* oviposition have been tested in the laboratory and in the field, for example: 1‐octen‐3‐ol, geosmin, or 2‐pentylfuran repel and reduce *D. suzukii* infestation under field conditions.^30^, ^33^, ^34^ Geosmin and 1‐octen‐3‐ol are volatile compounds originating from pathogen infested fruit^35^, ^36^ which can be a signal to *D. suzukii* that the substrate is not a viable feeding and oviposition site. *Drosophila suzukii* adults showed a reduced attraction to odours of raspberry fruit infected with *Botrytis cinerea*.^37^ The challenge in using volatile compounds as repellents in the field is their volatility in the environment which may reduce the time they remained effective. Habituation of *D. suzukii* to repellent odours due to prolonged exposure may also reduce effectiveness.^38^

Despite *D. suzukii*'s oviposition in underripe and ripe fruits, they also oviposit in damaged and rotten fruits especially towards the end of the commercial fruit growing season when ripening fruit are less available.^10^, ^39^, ^40^ At this time, *D. suzukii* are likely to encounter other ovipositing *Drosophila* species.^14^, ^15^ These interspecific interactions between two or more species can drive changes in distribution patterns and population dynamics of the species. Evidence has been gathered by several research groups to indicate these interspecific encounters are often detrimental to *D. suzukii*, resulting in negative implications for the population. Studies on the interactions between *D. suzukii* and the ‘model’ drosophila species, *Drosophila melanogaster* (Meigen), have been the main research focus, likely due to the existing wealth of knowledge surrounding *D. melanogaster* from its extensive use in research, ease of culturing and prevalence in most environments.^41^, ^42^

This mini review brings together recent works which focused on how *D. suzukii* egg laying and performance is affected by the presence of and their interaction with other *Drosophila* species. Understanding what factors determine the outcome of *D. suzukii* interactions with other *Drosophila* species and how this affects *D. suzukii* performance, feeding preference, or oviposition can be utilised to develop behavioural‐driven methods to reduce *D. suzukii* populations in the field.

## 2 INTERACTIONS BETWEEN *D. SUZUKII* AND OTHER *DROSOPHILA* SPECIES ON SHARED RESOURCES WITH THE FOCUS ON OVIPOSITION BEHAVIOUR

*Drosophila suzukii*'s propensity to oviposit on ripening fruits sets it apart from other species, where *D. suzukii* are less likely to face competition for shared egg laying resources. Morphologically with their serrated ovipositor, and behaviourally, *D. suzukii* prefer to lay eggs on ripening fruit.^43^, ^44^ *Drosophila suzukii* were also shown to prefer laying their eggs on spherical surfaces with smaller radius which mimic ripening fruits that are smaller in size than ripe fruits.^45^ Other closely related species to *D. suzukii*: *Drosophila subpulchrella* (Takamori & Watabe), *Drosophila mimetica* (Bock & Wheeler), and *Drosophila biarmipes* (Malloch) also possess a similar serrated ovipositor but with bristles and not as enlarged as *D. suzukii*.^15^ Out of these four closely related species, to date *D. suzukii* is the most significant threat to soft fruit production.

Previous visitation on a food source by other *Drosophila* species, such as *D. melanogaster* or *Drosophila lutescens* (Malloch) prior to the introduction of *D. suzukii* have consistently led to fewer eggs laid by *D. suzukii* compared to egg laying on control media not exposed to non‐*D. suzukii* species.^46^, ^47^ Comparable results were shown by Kienzle and Rohlfs where *D. suzukii* deposited significantly fewer eggs on blueberries previously oviposited on by *D. melanogaster* or wounded, indicating the presence of heterospecific cues by non‐*D. suzukii Drosophila* species, compared to intact and untouched blueberries that had never been visited by any flies.^48^ The general idea was that *D. suzukii* detected cues or signals left on the food source by non‐*D. suzukii* species which led to avoidance and ultimately deterred *D. suzukii* from ovipositing. However, the identity of these cues or signals remains unclear and as such it is not yet established if *D. suzukii* detects interspecific signals left on the media or fruit from other Drosophila species where the outcome is *D. suzukii* deterrence.

From a pest management perspective, studies to determine the identity of *D. suzukii* oviposition deterrent signals which originated from another *Drosophila* species will be of interest to be explored and potentially incorporated into formulations of *D. suzukii* oviposition repellents as shown previously using microbe‐ and pathogen‐derived compounds such as geosmin, 1‐octen‐3‐ol, and 2‐pentylfuran.^30^, ^33^, ^34^ If a *Drosophila*‐derived oviposition repellent to *D. suzukii* can be identified, a future research direction can be to artificially synthesise and deploy such oviposition repellent compounds in crops to deter *D. suzukii* from laying eggs.

Not all interactions with other *Drosophila* species negatively impact *D. suzukii* oviposition. Certain interspecific interactions have been reported to have no effect on *D. suzukii* oviposition. For example, *D. suzukii* exhibited no oviposition avoidance on food substrates previously exposed to *Drosophila rufa* (Kikkawa & Peng) or *Drosophila auraria* (Peng).^49^ This does not rule out the possibility that physical changes on the egg laying media caused by feeding and egg laying activities by other *Drosophila* species were the cause of *D. suzukii* oviposition avoidance. Substrate hardness, fruit firmness, and skin penetration force influence *D. suzukii* oviposition preference where firmer substrates are the preferred oviposition site.^50^, ^51^

When adults of both *D. suzukii* and *D. melanogaster* were contained together on the same food source, there were approximately six times fewer *D. suzukii* offspring per female compared to *D. melanogaster* ^52^ and fewer *D. suzukii* larvae survived through to pupation.^53^ At high population densities, *D. suzukii* larval mortality increases, subsequently decreasing the rate of adult emergence, both in the presence of their conspecifics or other species.^54^, ^55^ It has been suggested that this was the result of *D. suzukii* eggs being predated by *D. melanogaster* larvae.^52^ In Drosophila, when one more species shared the same food source, larval predation by another species or by its conspecifics were more likely to occur at high population densities, on low nutrient food, or when larvae had been starved.^56^, ^57^ In laboratory settings, *D. melanogaster* presence or previous presence on the food source reduced the *D. suzukii* population^46^, ^47^, ^52^ but it is not clear whether this finding can be utilised and translated into practice, as fruit infestation by *D. melanogaster* in a commercial setting would be undesirable. Although, in theory, the presence of *D. melanogaster* in fallen fruits may reduce *D. suzukii* emergence through competition and may indirectly aid control efforts, sanitary practices are vital as fallen fruits may attract further pests and pathogens. Though pest managers will not use *D. melanogaster* to reduce *D. suzukii*, studies on microbes and chemical cues left by *D. melanogaster* which mediate this interaction could be utilised to reduce *D. suzukii* oviposition.

Therefore, although studies to investigate the impact of larval predation and competition by *D. melanogaster* in reducing *D. suzukii* adult emergence are not directly relevant to pest management, it may be possible to utilise the knowledge derived in developing methods to reduce *D. suzukii* oviposition on fruits. Studies to determine if *D. melanogaster* in or on fruit reduce *D. suzukii* populations due to chemicals or compounds secreted by *D. melanogaster* would be of interest. This has potential practical applications, if it were possible to identify and synthesise the compounds involved, to deter *D. suzukii* in the field.

*Drosophila suzukii* experienced greater larval mortality in the presence of other fly species. For example, Pfeiffer and co‐workers recorded higher larval mortality of *D. suzukii* in grapes in the presence of African fig fly larvae, *Zapriounus indianus* (Gupta) but this depended on grape variety.^58^, ^59^ When cornmeal media was used as the egg laying substrate instead of grapes, *Z. indianus* presence did not affect *D. suzukii* mortality suggesting that food type had an impact on the outcome of this interspecific interaction.^58^, ^59^

The presence or previous presence of other *Drosophila* species can impose various effects on *D. suzukii* oviposition and this is governed by the type of media or fruit, fruit variety, length of exposure, and the interacting species. Oviposition behaviour of *D. suzukii* and other insects are determined by multiple factors where interspecific interaction is only one of them. This knowledge can potentially be useful to estimate the severity of *D. suzukii* infestation in the crop. For example, based on the presence and abundance of other Drosophila species which outcompete *D. suzukii*. Interspecific interactions are governed by multiple factors. In the following section, the role of microbes and cuticular hydrocarbons will be described and their potential applications as control methods highlighted.

## 3 THE ROLE OF MICROBES IN AFFECTING *D. SUZUKII* BEHAVIOUR AND INTERSPECIFIC INTERACTIONS

Microbes, including bacteria and fungi (including yeast) on food sources, on and inside insect bodies and their digestive tracts influence insect behaviour and development.^60^, ^61^, ^62^ Specific microbes are commonly found in *Drosophila*. For example, the yeasts *Hanseniaspora uvarum* (Niehaus) and *Pichia kluyveri* (Bedford ex Kudrjanzev) and the bacteria *Acetobacter* spp.^63^, ^64^, ^65^ are associated with both *D. suzukii* and *D. melanogaster*. The composition of *Drosophila* gut microbiota varies depending on food source and rearing condition,^66^, ^67^, ^68^ which differ between different species and different populations of the same species.^62^ Gut microbes in *Drosophila* are involved in kin recognition,^69^ for example: female *D. suzukii* were attracted to symbiotic acetic acid bacteria isolated from their conspecifics^70^ and microbial‐derived volatiles isolated from *D. suzukii*‐infested fruits exhibited varying attractiveness to wild populations of *D. suzukii*.^71^ Wild populations of *D. suzukii* were more attracted to *Gluconobacter* bacteria and *Hanseniaspora* yeast compared to *Pichia* sp. and *Curtobacterium* sp. yeast when used as baits in traps in raspberry fields.^71^ Baiting mixtures vary in their attractiveness to *D. suzukii* when deployed in the field and depending on the host fruit crop. Research is ongoing to optimise attractiveness of baiting and trapping mixtures in different crops.

Several laboratory studies have demonstrated that *Drosophila*‐associated microbes can influence the outcome of the interspecific interaction between *D. suzukii* and other *Drosophila* species. *Drosophila suzukii* laid fewer eggs on media inoculated with microbes isolated from *D. melanogaster* and *D. biarmipes*.^72^ In addition, microbes isolated from *D. suzukii* can influence *D. melanogaster* larval development.^73^ Compared to wild *Drosophila* populations, laboratory‐reared *Drosophila* had less diverse microbial communities due to their less varied diet and higher likelihood of population bottlenecks.^66^

*Drosophila* gut microbes influence the composition of their faecal matter or frass.^74^, ^75^ *Drosophila melanogaster* exhibited varying degrees of attraction to odour pulses generated from frass collected from several different Drosophila species.^75^ *Drosophila suzukii* females laid fewer eggs on blueberry fruits which were exposed to, fed upon, and contaminated with faecal matter from non‐*D. suzukii Drosophila* species.^48^ This demonstrated the likely involvement of microbes in *Drosophila* interspecific interactions. Responses to *Drosophila* associated microbes are highly variable, particularly when microbial‐based controls are deployed in the field hence pest control strategies using microbes remains challenging. For field application, delivery method and stability of microbes remains an area to be optimised.

Different species of yeast have been isolated from different *Drosophila* species.^76^ The flies' diet is a major determinant for *Drosophila*‐associated yeast species composition and yeast is a main food source.^67^ *Drosophila suzukii* were attracted to yeasts on food sources^77^ and laid more eggs on food containing yeasts.^49^ It is worth noting that laboratory‐based work showed that presence or absence of other *Drosophila* species as competitors can change *D. suzukii*'s preference for yeast‐supplemented food and the effect is species‐specific.^49^ To incorporate this into pest management programmes, the application of a sterilant on fruit surfaces reduced *D. suzukii* settlement, growth, and egg‐laying in both laboratory‐ and in field‐based experiments.^72^ More specifically, when a sterilant composed of peroxyacetic acid and hydrogen peroxide was applied in the field, this led to lower infestation of *D. suzukii*. In the laboratory, *D. suzukii* growth were inhibited on food sources treated with crop sterilant and impacted *H. uvarum* and *Issatchenkia terricola* (Van der Walt); yeasts known to be attractive to *D. suzukii*.^72^ Using sterilants to alter the composition of yeast or microbes present on the crop can be incorporated into integrated management for *D. suzukii* but optimisation is needed depending on the particular mixture of sterilant, yeast, and fruit crops.

Different yeast species exerted varying degrees of attractiveness to different *Drosophila* species and these can influence fly behaviour.^76^, ^78^, ^79^, ^80^ *Drosophila suzukii* laid more eggs on cherry fruits colonised by *Candida* sp. and *Saccarhomyces cerevisiae* (Meyen ex. EC Hansen).^81^ Yeast‐derived volatiles from fermenting fruits promoted *D. melanogaster* feeding and oviposition^82^ and *D. melanogaster* larvae were attracted to yeast species known to improve larval fitness.^83^ However, it is not yet clear whether certain yeast species provide specific fitness benefits or whether the response is species‐specific for both *Drosophila* and the yeast species they interact with.^81^, ^84^

*Drosophila* can distinguish different yeasts. *Drosophila suzukii* and *D. melanogaster* can discriminate volatiles between six different yeast species and exhibited distinct olfactory responses to yeast‐derived volatiles.^85^ The ability of *D. suzukii* to detect specific yeast‐derived volatiles coupled with the knowledge that the presence or absence of yeast influence the attractiveness of food and oviposition source makes yeast a useful tool for incorporation into the development of *D. suzukii* push–pull control systems.

Adult and larval stages of *D. suzukii* showed a preference for the yeast *H. uvarum* on food sources.^85^, ^86^, ^87^, ^88^ *Hanseniaspora uvarum* also increases the fecundity of female *D. suzukii*.^89^ *Drosophila suzukii* female laid more eggs on *H. uvarum*‐inoculated blueberries and are more attracted to *H. uvarum* volatiles compared to volatiles of *S. cerevisiae* or *Pichia terricola*.^88^ Utilising this preference, *H. uvarum* yeast has been combined with insecticides as part of successful attract and kill approaches to reduce *D. suzukii* populations.^90^, ^91^, ^92^ This strategy can reduce the amount of insecticide needed to reduce *D. suzukii* populations in the field, leading to significant cost reduction for growers and reduction in fruit insecticide residues.^91^, ^93^

There are challenges in utilising and incorporating yeast and/or microbial volatiles into developing methods to reduce *D. suzukii* population in the field. Firstly, laboratory‐reared versus wild‐caught *D. suzukii* can respond differently to certain microbes. Secondly, different outcomes can be observed when testing individual or blends of microbes.^1^, ^31^, ^71^, ^94^ However this is not always the case whereby a study showed that both individual or the combination of metabolic volatiles from two yeast species *H. uvarum* and *Metschnikoiwa pulcherrima* were attractive to *D. suzukii*.^87^ Large‐scale testing is required to determine the most effective microbial‐derived blends and their longevity in the field.

Identifying and utilising microbial blends to increase the selective attraction of *D. suzukii* over other *Drosophila* species is ongoing, particularly in trap development^95^, ^96^, ^97^, ^98^ to reduce the time and labour to identify *D. suzukii* trap catches.^99^ Whilst it has been shown that microbes, either present on food sources or those that form close association with the flies themselves can influence their behaviour, applications to utilise microbe‐derived control methods remain challenging. This is mainly due to multiple aspects influencing both flies‐associated microbial community and their responses to it. More specifically, challenges remain in optimising delivery methods, dosage, stability, and longevity of microbes once deployed in the environment. Studies to understand how microbes may influence other insects or natural enemies is also vital to consider.

Lastly, it has been proposed that *Drosophila*‐associated microbes can influence *Drosophila* cuticular hydrocarbons (CHCs) and this is influenced by their diet.^100^ The role of CHCs in *Drosophila* communication and interaction will be covered in the next section.

## 4 *DROSOPHILA* CUTICULAR HYDROCARBONS AND THEIR ROLE IN INTERSPECIFIC INTERACTIONS BETWEEN *D. SUZUKII* AND OTHER *DROSOPHILA* SPECIES

Interspecies and intraspecies communication between *Drosophila* are mediated via multiple signals including insect CHCs. The majority of CHCs are low in volatility and detected by insects via short‐distance communication^101^ or through direct physical encounters. CHCs can be detected in the air (headspace), on the bodies, or on surfaces previously visited by insects.^102^ CHCs are implicated in *Drosophila* courtship and mating behaviour,^102^ and function as sex pheromones^103^ and in species recognition.^104^, ^105^ Gender, age, mating status, and geographical location can influence *Drosophila* CHCs.^101^, ^105^, ^106^

The role of CHCs in interspecific interactions between *D. suzukii* and other *Drosophila* species is an area worthy of further investigation. A recent paper showed that presence of live *D. melanogaster* larvae led to reduced egg laying by *D. suzukii* but there was no evidence for the involvement of CHCs in this.^47^

The widely studied male *D. melanogaster* sex pheromone, *cis*‐11‐vaccenyl acetate (*c*VA) is not a CHC but has multiple roles including promoting the aggregation of conspecifics,^107^ increasing mating acceptance by females,^108^ suppressing courtship by other males,^109^, ^110^ and increasing aggression between conspecific males.^111^ Perfuming male *D. suzukii* with *c*VA disrupted mating^112^ which indicated that *D. suzukii* detect and respond to *c*VA, even though *D. suzukii* does not produce it.

Disruption of the relative amounts of four major CHCs (Z)‐5‐, (Z)‐7‐, (Z)‐9‐tricosenes and tricosane also led to mating and courtship disruption in *D. suzukii*.^105^ One of these CHCs, the 9‐tricosene, has been patented (patent number EP 34557846) for use in *D. suzukii* pest management.^113^ The identification of behaviour‐altering *Drosophila* CHCs can be utilised in developing a push–pull based strategy to protect crops from *D. suzukii* but effective formulation and field application of this still needs further study.^114^ The prospect of identifying, synthesising, and utilising CHCs derived from other *Drosophila* species to deter oviposition by *D. suzukii* have been suggested but not fully explored to date.^46^, ^47^, ^52^, ^53^

*Drosophila* can detect CHCs and sex pheromone such as *c*VA from their conspecifics and those of other species resulting in behavioural changes. CHCs from adult *Drosophila simulans* and *Drosophila buzzatii* (Patterson and Wheeler) repelled larvae of *D. simulans* from a food source.^115^ Larvae of *D. melanogaster* detect conspecific cues demonstrated by more frequent visits to patches of eggs from their kin compared to non‐kin eggs.^116^ Female *D. melanogaster* laid more eggs where their conspecific larvae were present^117^ and *D. melanogaster* larvae were attracted to food odours previously consumed by conspecifics.^118^ It is not yet confirmed if the same response is characteristic of *D. suzukii* larvae in the presence of their conspecifics or whether this observed effect is mediated by *Drosophila* CHCs. Studies to investigate how *D. suzukii* adult flies and larvae respond to CHCs from conspecifics and from non‐*D. suzukii* species would enable a greater understanding of the role of CHCs in influencing the outcome of interspecific interactions and understanding the ecology of *D. suzukii*. Utilising the knowledge gained from CHCs in behavioural‐driven integrated control methods for *D. suzukii* remains to be developed and optimised. Similar to microbes, questions remain on delivery methods, stability in the environment, compatibility with other integrated pest management methods, and impact on other insects in the environment.

## 5 CONCLUSIONS

*Drosophila suzukii* oviposition and adult emergence generally decline when sharing a food source with other *Drosophila* species. Studies to investigate whether the decline in *D. suzukii* oviposition and offspring emergence is due to an insect‐derived signal (e.g., pheromone, CHCs), or microbial‐derived source (e.g., *Drosophila* microbiome, food‐associated or fruit microbiome^119^) have only just begun to reveal in more depth what drives *D. suzukii* behaviour. The mechanisms underlying the observed oviposition deterrent effect to *D. suzukii* during presence or previous exposure of media to non‐*D. suzukii* species, the origin of the signal involved, and how this is detected by *D. suzukii* are not fully understood yet and this opens new areas of research.

Knowledge on how *D. suzukii* perceive signals originated from other *Drosophila* species, for example, via olfaction or gustation, and how this alters or deters *D. suzukii* oviposition would be useful to aid in signal identification, formulation, and if appropriate, to design a suitable device to deploy synthesised compounds for field application to reduce *D. suzukii* populations. Studies reviewed here demonstrated that previous presence or presence of live larvae or adults of *D. melanogaster* deter *D. suzukii*.^46^, ^47^, ^52^ This implies that a signal or cue from *D. melanogaster* is detected by *D. suzukii*, which warrants further research in the field.

The use of insect‐, microbial‐, or plant‐derived compounds to deter *D. suzukii* oviposition on fruits would be favourable to reduce the use of more conventional plant protection products and to minimise the risk of resistance developing. For example, plant‐derived extracts from the plant genera *Actinidia*, 1‐octen‐3‐ol which is a microbe‐produced mushroom alcohol, geosmin, or 2‐pentylfuran deter *D. suzukii* oviposition in a laboratory setting.^31^, ^33^, ^34^, ^119^

There is a significant impact of using different *D. suzukii* populations, substrate types and microbial communities in laboratory and field tests leading to variation in experimental results. In addition, not all pre‐exposure of media to non‐*D. suzukii Drosophila* species elicit an oviposition deterrent effect in *D. suzukii*. A useful avenue of research would be to determine, in a more ecological setting, how *Drosophila* species in a community interact and under what conditions. Most studies have been done in rather artificial laboratory settings, on substrates or fruit out of context of the surrounding habitat and environment. The outcome of microbial‐derived cues in influencing *D. suzukii* behaviour is context‐dependent and involves multiple factors, such as the blend and composition of the microbial species, the flies' sex and different developmental stages, and other stimuli present in the environment. *Drosophila suzukii* uses multiple cues to determine their feeding and oviposition site, where these cues can have either additive or synergistic effects.^114^ Development of an effective integrated pest management method to manage *D. suzukii* population needs a thorough understanding of these multiple factors influencing their behaviour and fitness.

Control practices target wild populations of *D. suzukii* and therefore seasonal preferences and dimorphism of *D. suzukii* need to be considered.^120^ At different times of the year and depending on temperature, *D. suzukii* are present as either winter or summer morphs.^121^ Several studies have demonstrated that *D. suzukii* respond to different cues depending on their morphological state. Summer and winter morphs of *D. suzukii* displayed different responses to blends of yeast volatiles^122^ and semiochemical repellents.^32^ Other studies showed that strawberry fruit volatiles were more attractive to summer morphs,^123^ and yeast‐based lures were more attractive to winter morphs.^124^ These differences in behavioural pattern between seasonal morphs of the flies are most likely driven by availability and abundance of food source in the environment at different times of the year. Understanding the behavioural responses of different *D. suzukii* seasonal morphs to specific volatile organic compounds or baits is needed to optimise year‐round pest management strategies. There is no investigation, to date, on *D. suzukii* winter morphs in response to other *Drosophila* species.

Beside seasonal dimorphism, research into genetic diversity and population dynamics of *D. suzukii* will provide a better understanding of their ecology and enable this knowledge to be incorporated in integrated pest management strategies. Several studies have explored genetic variation of *D. suzukii* in the United States, Germany, and Italy.^125^, ^126^, ^127^ There are no studies yet to determine whether behavioural changes in *D. suzukii*, driven by its interactions with other *Drosophila* species, vary between genetically distinct populations of *D. suzukii*.

This review covers factors that influence interspecific interactions, focusing on microbes and CHCs and their effect on *D. suzukii* oviposition. Research pathways to incorporate these interspecific factors into applied, behaviour‐driven control methods for *D. suzukii* are still ongoing, particularly focussing on delivery methods, stability in the environment, and impact to other species and natural enemies. Only by understanding wider interspecific interactions in a more ecologically relevant setting will we be able to translate fundamental research into practice and apply such findings to develop effective integrated pest management strategies manipulating *D. suzukii* behaviour.

## CONFLICT OF INTEREST STATEMENT

The authors declare no conflict of interest.

## Data Availability

This manuscript is a mini review. Therefore the data presented in this manuscript are a collation of published and peer‐reviewed manuscript relevant to the topic of this mini review.
